# Different regimens of penicillin antibiotics given to women routinely for preventing infection after cesarean section: A systematic review and meta analysis

**DOI:** 10.1097/MD.0000000000011889

**Published:** 2018-11-16

**Authors:** Dan Liu, Lingli Zhang, Chuan Zhang, Min Chen, Li Zhang, Jinke Li, Guanjian Liu

**Affiliations:** a Department of Pharmacy/Evidence-Based Pharmacy Center, West China Second University Hospital; b Key Laboratory of Birth Defects and Related Diseases of Women and Children; c Department of Obstetrics and Gynecology, West China Second University Hospital; d Cochrane China, West China Hospital, Sichuan University, Chengdu, China.

**Keywords:** cesarean section, infection, penicillin antibiotics, systematic review

## Abstract

**Background:**

Varied regimens of penicillin antibiotics were given to women for preventing infection after cesarean section, but there is no study compares the effectiveness and safety of them.

**Methods:**

We searched MEDLINE, Embase, CENTRAL, CNKI, Wanfang, VIP, and CBM Database, and contacted experts in the field and searched reference lists of retrieved studies. We included randomized controlled trials comparing different regimens of penicillin antibiotics given to women after cesarean section. Two review authors independently assessed the studies for inclusion, assessed risk of bias, and carried out data extraction.

**Results:**

A total of 18 randomized controlled trails (involving 3287 pregnant women) were eligible. Compared with after umbilical cord clamping, penicillin antibiotics prophylaxis before skin incision could reduce the risk of endometritis for women undergoing cesarean. Compared with using penicillin antibiotics alone, using antibiotic–inhibitor combination could reduce the risk of endometritis or fever. No statistically significant difference was present between single-dose versus multidose, short term versus long term, intravenous injection versus lavag in the risk of reported outcomes.

**Conclusion:**

There is insufficient evidence to draw certain conclusions on which regimen of penicillin antibiotics is the best in this review. Further studies should pay attention to the study design, and besides the outcomes of pregnant women, researchers should focus on the outcomes of newborns.

## 1 Introduction

A cesarean section is a very common surgical operation in the obstetric. Reports have shown that rates of cesarean sections have increased above 15% in approximately half of countries worldwide.^[1]^ However, a high rate of cesarean sections means high maternal and neonatal risk. The risk of postpartum infection of cesarean sections is nearly 5 times as vaginal births, and cesarean sections are associated with more neonatal respiratory morbidity and sepsis than those delivered by normal vaginal delivery.^[2,3]^ It has been proven that compared with no prophylactic antibiotics, prophylactic antibiotics in cesarean section contributes to reducing the risk of the incidence of febrile morbidity, wound infection, optometrists, and serious maternal infectious complications.^[4]^ According to a Cochrane review, both the phosphorescence and penicillins represent good choices for prophylaxis in women undergoing cesarean section.^[5]^ At present, there are varied regimens of penicillin antibiotics given to women for preventing infection after cesarean section. However, there is no study that compares the effectiveness and safety between the specific subclasses of penicillins, or the administration timing, frequency, and route of penicillin antibiotics. Thus, we are unable to choose the ideal regimen of penicillin antibiotics for cesarean section based on the best available evidence. Therefore, we intend to undertake this review to compare different regimens of penicillin antibiotics given to women routinely for preventing infection after cesarean section. In this review, we did not only compare different penicillin antibiotics but also the administration regimens including different doses, different intervals, and different courses.

## 2 Methods

### 2.1 Eligibility criteria

We included randomized controlled trials investigating maternal and fetal outcomes of prophylactic penicillins regimens for women undergoing cesarean section. The types of participants were women undergoing cesarean section. The types of interventions including the comparison between 2 or more different kinds, doses, administration intervals, and courses of treatment of penicillin antibiotics. The primary outcomes were sepsis in the mother and/or infant, endometritis, infant oral thrush; secondary outcomes were febrile morbidity, urinary tract infection, wound infection, adverse events of treatment on both mother and infant, and maternal length of stay and cost. We did not apply any language restrictions. We excluded those reported as abstracts only due to limited information.

### 2.2 Literature search

The search strategy of this review was designed by the Cochrane Pregnancy and Childbirth Group. We searched the Cochrane Central Register of Controlled Trials (CENTRAL), MEDLINE (Ovid), Embase (Ovid), CINAHL (EBSCO), CNKI Database, Wanfang Database, VIP Database, and CBM Database for studies published before February 2018. We hand-searched journals and conference proceedings of major conferences. Search terms are ((penicillins [mesh] OR penicillins [text word] OR amoxicillin [mesh] OR amoxicillin [text word] OR ampicillin [mesh] OR ampicillin [text word] OR piperacillin [mesh] OR piperacillin [text word] OR azlocillin [mesh] OR azlocillin [text word] OR mezlocillin [mesh] OR mezlocillin [text word] OR dicloxacillin [mesh] OR dicloxacillin [text word] OR flucloxacillin[mesh] OR flucloxacillin [text word] OR cloxacillin[mesh] OR cloxacillin [text word] OR carbenicillin[mesh] OR carbenicillin [text word] OR ticarcillin[mesh] OR ticarcillin [text word] OR nafcillin[mesh] OR nafcillin [text word]) AND (cesarean section [mesh] OR cesarean section [text word]).

In addition, we contacted authors/experts in the field for unpublished and ongoing trials, and we also checked the reference lists of retrieved studies.

### 2.3 Selection of studies

Two review authors (DL and MC) independently screened titles and abstracts for potentially eligible studies; and read full texts for final eligibility. If disagreement occurred, the eligibility was decided by the third person (LZ).

### 2.4 Data extraction and management

We designed a form to extract data including the following information: details of source, eligibility, methods, participants, interventions, outcomes, and results. For eligible studies, 2 review authors (DL and CZ) extracted the data independently. The discrepancies were resolved by consulting a third author (LZ). When information regarding any of the above is unclear, we attempt to contact the authors of the original reports to provide further details.

### 2.5 Risk of bias assessment

Two review authors (LZ and JL) independently assessed risk of bias for each study using the criteria outlined in the Cochrane Handbook for Systematic Reviews of Interventions. The disagreement was resolved by involving a third assessor (GL).

### 2.6 Data analysis

For dichotomous data, we presented results as summary risk ratio with 95% confidence intervals; for continuous data, we used the mean difference or standardised mean difference with 95% confidence intervals. Fixed-effect meta-analysis was used for combining data or, in the event of statistically significant heterogeneity (*I*^*2*^ > 30%) between estimates, random effect models. If we identify substantial heterogeneity, we investigated it using subgroup analyses and sensitivity analyses. If there are 10 or more studies in the meta-analysis, we would investigate reporting biases using funnel plots. We carried out statistical analysis using the Review Manager software (RevMan 2014).

## 3 Results

### 3.1 Search results

**Figure 1:**
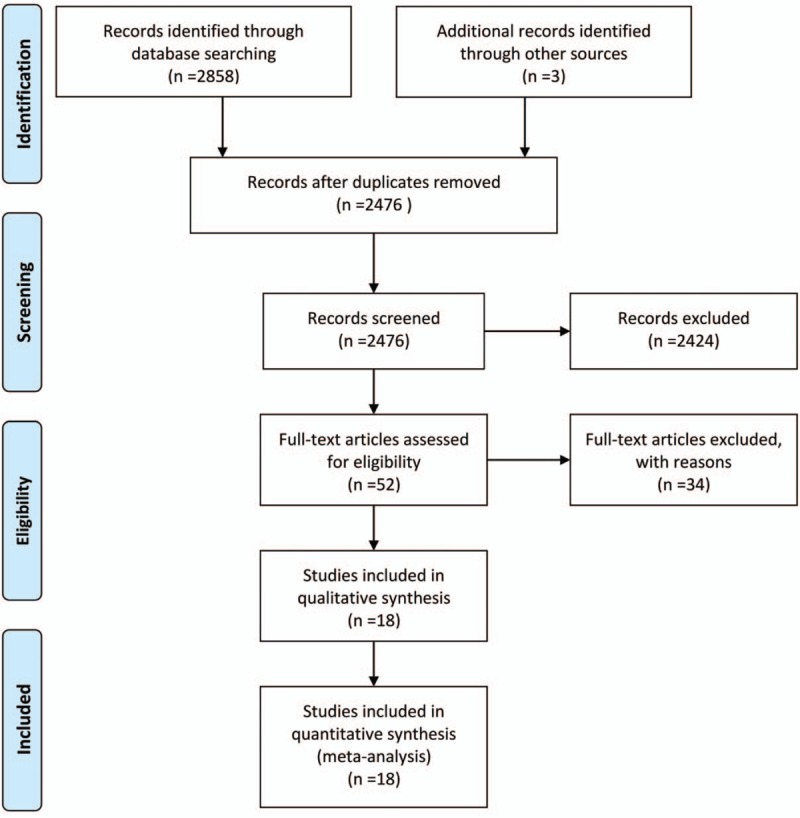
Flow diagram of study selection process.

The search in electronic databases yielded 2858 citations, and further 3 studies were identified through other sources (Fig. 1). We included 18 studies involving 3287 women. Of those 18 eligible studies, 3 were conducted in Europe, 6 in North America, and 9 in Asia (Table 1). The studies enrolled 60 to 432 women with mean age ranging from 21.6 to 28.5 years old. The penicillins used in these studies including ampicillin, ampicillin-salbactam, mezlocillin, piperacillin, piperacillin-tazobactam, amoxyicillin, and amoxycillin-clavulanic acid. The comparisons were single dose versus multidose, short term (<24 hours) versus long term (>24 hours), before skin incision and after umbilical cord clamping, intravenous injection versus lavage, antibiotic combined inhibitor with versus antibiotic alone. The studies only reported maternal outcomes including sepsis, endometritis, fever, urinary tract infection, wound infection, febrile morbidity, length of stay and costs, adverse events.

**Table 1:**
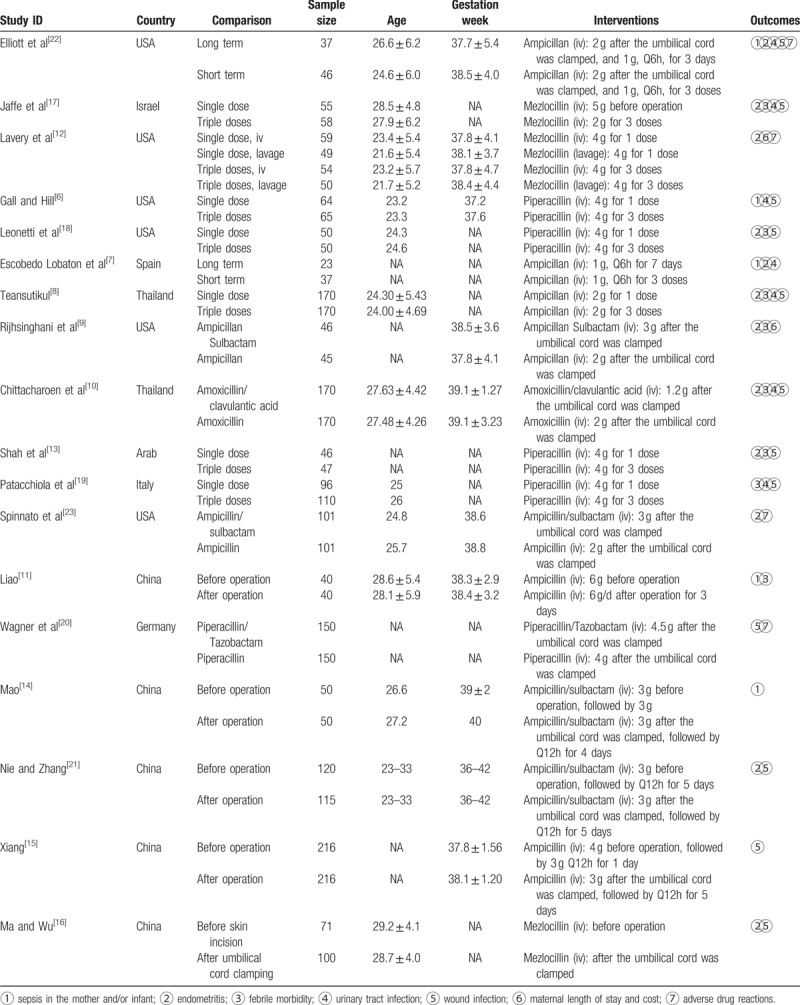
Characteristics of the included studies.

### 3.2 Risk of bias of eligible studies

Table 2 provides detailed information on risk of bias. All included studies stated they were randomized controlled trials, but only 5 studies described the specific method of generating the randomization sequence,^[6–10]^ and 1 study used the wrong method of generating the randomization sequence.^[11]^ Five studies had adequate descriptions of allocation concealment,^[8–10,12,13]^ with the remaining trials assessed as unclear risk. Two studies described appropriate methods for blinding of participants, personnel, and outcome assessors,^[9,10]^ and 5 studies^[8,11,14–16]^ were not blinding studies. There was no risk of attrition bias due to exclusions or withdrawals in all studies. There was no evidence of selective reporting for 10 included studies.^[9,11–17,21,22]^ One trial was at high risk of reporting bias for not reporting outcome results according to treatment arm.^[23]^

**Table 2:**
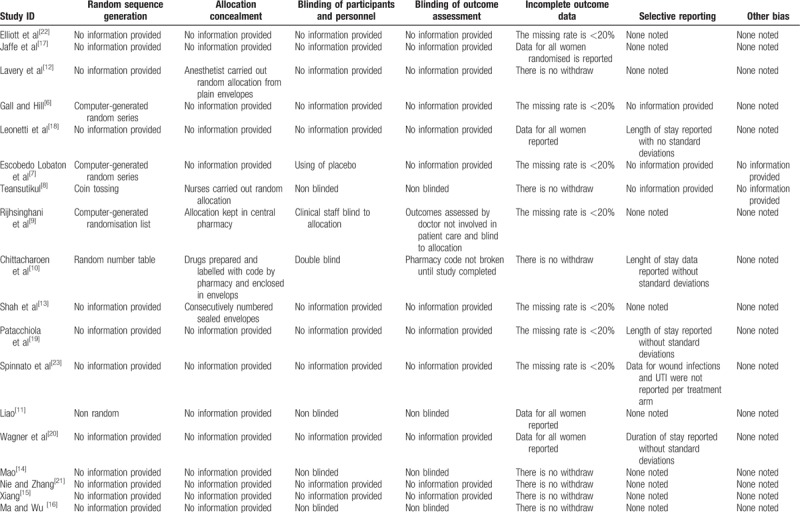
Risk of bias of included studies.

### 3.3 Synthesis of results

#### 3.3.1 Single dose versus multidose

Seven studies^[6,8,12,13,17–19]^ involving 1180 pregnant women compared the single dose and multidose. The outcomes reported in these studies are maternal sepsis, endometritis, fever, urinary tract infection, wound infection, and length of stay. One study reported maternal sepsis as an outcome and showed no statistically significant difference between single dose and multidose administration in maternal sepsis (RR 3.05, 95% CI 0.13 to 73.41; participants = 129).^[6]^ Five studies reported endometritis as an outcome and showed no statistically significant difference between single dose and multidose administration in endometritis (RR 1.05, 95%CI 0.70 to 1.60, *I*^2^ 0%, participants = 759).^[8,12,13,17,18]^ Five studies reported the outcome maternal febrile morbidity, and the result showed no significant difference between single-dose and multidose administration in maternal febrile morbidity (RR 1.05, 95% CI 0.72–1.52, *I*^2^ = 0%; participants = 859).^[8,13,17–19]^ Four studies reported the outcome of urinary tract infections, and showed no significant difference between single-dose and multidose in reducing urinary tract infection rate in mother (RR 0.61, 95% CI 0.25–1.49, *I*^2^ = 0%; participants = 775).^[6,8,17,19]^ Six studies reported wound infection in mother, and showed no significant difference between single dose and multidose administration in wound infection rate in mother (RR 0.81, 95% CI 0.30–2.25, *I*^2^ = 0%; participants = 988).^[6,8,12,17–19]^ One study reported length of stay in mother, involving 113 patients, and showed that no significant difference between single-dose and multidose administration in length of stay (MD 0.60, 95% CI −0.28– 1.48; participants = 113).^[12]^ Three studies reported the outcome of adverse effects of treatment. Two trials reported zero events in each arm and therefore did not contribute to the analysis.^[6,18]^ Shah et al^[13]^ reported 1 broncospasm (single-dose group) and 1 skin reaction (three-doses group), so that there was no difference in the rate of adverse events between the treatment groups (RR 1.08, 95% CI 0.07–16.84; participants = 316).

#### 3.3.2 Short term versus long term

Two studies involving 143 pregnant women compared short term and long term.^[7,22]^ The outcomes reported in these studies are endometritis, fever, urinary tract infection, and wound infection. Elliott et al^[22]^ involving 83 patients, compared the effect of one-day penicillin antibiotics with three-day penicillin antibiotics. There was no evidence of difference between the one-day and three-day groups in rates of maternal sepsis (RR 4.04, 95% CI 0.20–81.69), urinary tract infection (RR 2.43, 95% CI 0.10–57.86) and incision infection in mother (RR 2.43, 95% CI 0.10–57.86). Use of three-day course of antibiotics probably reduced the rate of endometritis in the mother, though this result is based on a small sample size (RR 3.49, 95% CI 1.07–11.33). The authors reported no adverse effects of treatment in either group, so the risk ratio could not be calculated. Escobedo Lobaton^[7]^ involving 60 patients, compared the effect of one-day penicillin antibiotics with 7-day penicillin antibiotics. There were no events in either arm for these outcomes, and so the risk ratio could not be calculated for either endometritis or sepsis. There was only 1 urinary tract infection, in the one day treatment group. This difference did not reach statistical significance due to too few events and too small sample size (RR 0.21, 95% CI 0.01–4.96).

##### 3.3.2.1 Timing

Five studies^[11,14–16,21]^ involving 1018 pregnant women compared the timing of administration. The outcomes reported in these studies are sepsis, endometritis, fever, and wound infection in mothers. Two studies reported sepsis as an outcome, and the pooling of data showed no statistically significant difference between administration before skin incision and after umbilical cord clamping in sepsis (RR 0.67, 95%CI 0.20–2.26, *I*^2^ = 0; participants = 180).^[11,14]^ Two studies reported endometritis as an outcome, and the pooling of data showed administration before skin incision could reduce the rate of endometritis (RR 0.21, 95%CI 0.10–0.45, *I*^2^ = 0; participants = 406).^[16,21]^ One study reported fever morbidity as an outcome, and showed no statistically significant difference between before skin incision and after umbilical cord clamping in febrile morbidity (RR 0.78, 95% CI 0.32–1.88; participants = 80).^[11]^ Three studies reported wound infection as an outcome, and the pooling of data showed administration before skin incision could reduce the rate of wound infection (RR 0.21, 95%CI 0.09–0.50, *I*^2^ = 0; participants = 838).^[15,16,21]^

##### 3.3.2.2 Administration route

One involving 108 pregnant women compared the effect of intravenous injection versus lavage.^[12]^ There was no evidence of difference between intravenous injection and lavage groups in rates of endometritis in the mother (RR 1.02, 95% CI 0.38–2.70), and no difference between intravenous injection and lavage groups in length of stay (MD: 0.40, 95% CI: −0.55–1.35).

### 3.4 Penicillins combined with inhibitor versus penicillins alone

Four studies involving 933 pregnant women compared the effect of penicillins combine with enzyme inhibitors with penicillins alone.^[9,10,20,23]^ The penicillins used in these studies are ampicillin (/sulbactam), piperacillin (/tazobactam) and amoxycillin (/clavulanic acid). The outcomes reported in these studies are endometritis, fever, urinary tract infection, and wound infection in mothers. Three studies reported endometritis as an outcome and showed that combined treatment with an inhibitor probably led to fewer cases of endometritis (RR 0.39, 95% CI 0.21–0.72, *I*^2^ 0%; participants = 633).^[9,10,23]^ Two studies reported on the outcome of maternal febrile morbidity and results from the meta-analysis showed use penicillins with inhibitor probably led to modest improvements in the rate of maternal febrile morbidity, though this result is based on small sample size (RR 0.46, 95% CI 0.21–1.00, *I*^2^ = 0%; participants = 431).^[9,10]^ One study reported the outcome of urinary tract infection in mother, with no evidence of group differences (RR 1.00, 95% CI 0.20–4.88; participants = 340).^[10]^ Results from the meta-analysis of 2 studies that reported wound infection showed there was no difference in rates of wound infection (RR 0.78, 95% CI 0.19–3.12, *I*^2^ 23%; participants = 640).^[10,20]^ One small study found no evidence of a difference in the number of women requiring length of hospital stay > 4 days (RR 0.91, 95% CI 0.50–1.67; participants = 91).^[9]^ Two trials reported no adverse events in any treatment arm, and so the risk ratio was not possible to calculate.^[20,23]^

## 4 Discussion

Around 18 included studies (n = 3287) contributed data for the analysis of this review. We analyzed outcome data for 5 comparisons: single versus multidose; short term versus long term; before skin incision versus after umbilical cord clamping; intravenous injection versus lavage; and finally, antibiotic combined with inhibitor versus antibiotic alone. Across all comparisons, 6 included trials reported the penicillin antibiotics were well tolerated, with no adverse events in any treatment arm. Another trial reported one adverse event in each treatment arm (one broncospasm in the single-dose group and one skin reaction in the three-dose group). Around 7 trials contributed outcome data for the comparison of single versus three-dose regimen. There was no evidence of difference for any reported outcome, including sepsis in the mother, endometritis, fever in mother, urinary tract infection, incision infection, length of stay, and adverse effects. Two small studies compared short term versus long-term regimens with no power to detect group differences for any reported outcome, including sepsis, endometritis, urinary tract infection, incision infection, and adverse events. Five studies compared the timing of administration. The pooling of data showed administration before skin incision could reduce the rate of endometritis and wound infection, but there is no statistically significant difference between administration before and after operation in sepsis, and febrile morbidity. One study compared intravenous injection versus lavage, and showed no evidence of difference for reported outcomes, including endometritis and length of stay. The final comparison of combination antibiotic with inhibitor versus antibiotic alone had the most evidence available, but even so evidence was sparse for individual outcomes. The antibiotic–inhibitor combination probably reduced the number of women who had endometritis or febrile morbidity when compared to the antibiotic alone. Two trials reported no adverse events in either treatment arm. There was no evidence of difference for the outcomes of urinary tract infection, incision infection or maternal length of stay > 4 days.

To our knowledge this is the first systematic review assessing the benefits and harms of different regimens of penicillin antibiotics administration for the prevention of infection after cesarean section. Baaqeel and Baaqeel^[24]^ reported lower endometritis rates when preoperative cephalosporin antibiotics versus intraoperative administration, which is similar to the results about administration timing of penicillin antibiotics in this study. Also the recent guideline also recommended the prophylactic antibiotics be prior to skin incision.^[25]^ Although the guideline recommended the single dose rather than multiple dose, the current study^[26]^ showed an uncertain conclusion for a trend toward a lower incidence of urinary tract infection observed with multiple dose regimens of cephalosporin antibiotic prophylaxis. In this study, we found there is no significant different between single dose and multiple dose. However, because no results for newborns were observed, the results should still be cautious. For other results, there is still insufficient evidence in other researches,^[27]^ as well as in this study.

Individual analyses for all outcomes in all comparisons were based on few studies and few women, limiting our confidence in the stability and certainty of findings. Reporting of the review primary and secondary outcomes was especially poor for infant outcomes, without a single included trial reporting the review outcomes of infant oral thrush or adverse events in the infant. The risk of bias in most trials was low or unclear, with poor reporting of methods contributing to our uncertainty for the important domains of sequence generation, allocation concealment and blinding of clinical staff, women and outcomes assessors. There were few adverse events reported in 6 trials, but there was no information reported on the influence of treatments on newborns.

## 5 Conclusions

There is insufficient evidence to draw certain conclusions on which regimen of penicillin antibiotics is the best in this review. Further studies should pay attention to the study design, and besides the outcomes of pregnant women, researchers should focus on the outcomes of newborns.

## Acknowledgments

We would like to thank their affiliated institutions and organisations. We would like to thank Program for Yangtze River Scholars and Innovative Research Team in University (No. IRT0935) and Group of People with Highest Risk of Drug Exposure of International Network for the Rational Use of Drugs, China. Thanks also to editors and review from Medicine, and the experts from the Cochrane Pregnancy and Childbirth Group.

## Author contributions

**Conceptualization:** Lingli Zhang, Jinke Li.

**Data curation:** Chuan Zhang, Jinke Li.

**Formal analysis:** Dan Liu.

**Funding acquisition:** Lingli Zhang.

**Investigation:** Chuan Zhang, Min Chen.

**Methodology:** Dan Liu, Li Zhang, Guanjian Liu.

**Project administration:** Dan Liu, Li Zhang.

**Resources:** Chuan Zhang, Min Chen.

**Supervision:** Lingli Zhang.

**Validation:** Min Chen.

**Writing – original draft:** Dan Liu.

**Writing – review & editing:** Dan Liu, Lingli Zhang, Guanjian Liu.
